# MAPD: a probe design suite for multiplex ligation-dependent probe amplification assays

**DOI:** 10.1186/1756-0500-3-137

**Published:** 2010-05-21

**Authors:** Jizu Zhi

**Affiliations:** 1 Bioinformatics Core Facility, School of Medicine, Stony Brook University, Stony Brook, NY 11794, USA

## Abstract

**Background:**

Multiplex ligation-dependent probe amplification (MLPA) was originally described as an efficient and reliable technique for gene dosage or DNA copy number variation (CNV) analysis. Due to its low cost, reliability, sensitivity, and relative simplicity, MLPA has rapidly gained acceptance in research and diagnostic laboratories, and fills the gap between genome-wide analysis and single gene analysis. A number of new applications have been developed shortly after the introduction of MLPA, including methylation-specific MLPA (MS-MLPA), the use of MLPA in SNP genotyping, copy number analysis in segmentally duplicated regions, etc. However, probe design is time consuming and error prone. Recently software has been developed to help human genomic MLPA probe selection and optimization. For other genomes and MS-MLPA, probe design remains a challenge.

**Findings:**

This paper describes a number of new features added to the previous H-MAPD software, which include: 1) probe selection for MS-MLPA; 2) support of mouse and rat genomes; 3) a set of new stuffer sequences. In addition, a physical-chemical property verification tool was implemented to verify user defined probes.

**Conclusions:**

MAPD is a web-based tool which is freely available to non-commercial users. The previous H-MAPD software has been used by about 200 users from more than 30 countries. With the new features, the author hopes MAPD will bring more convenience to the MLPA community.

## Findings

Like the original software [1], MAPD supports both electrophoresis-based and bead-coupled MLPA platforms. The software accepts one or more DNA sequences in FASTA format. Users should specify the genome (human, mouse, rat) to be analyzed. Users will also specify the desired protocol (electrophoresis-based or bead-coupled) and other experiment parameters (See Additional file 1: MAPD input page).

### Probe selection

**Figure 1:**
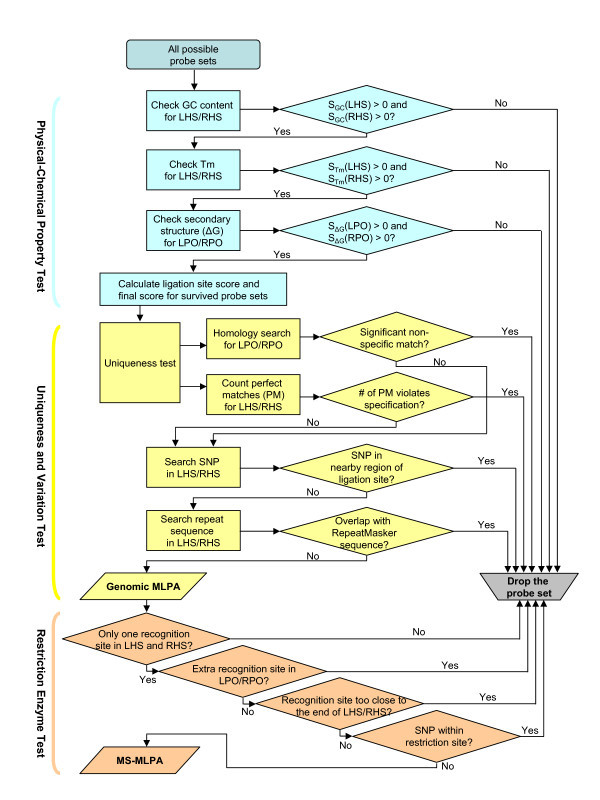
**Outline of probe selection workflow**. A successful genomic MLPA probe set needs to meet all the physical-chemical property criteria, and pass the uniqueness and variation test. For MS-MLPA, the probe sets need to meet all the restriction enzyme filtering criteria.

For genomic MLPA probe screening, the workflow is separated into two processes: 1) physical-chemical property test of hybridizing sequence and oligos; and 2) sequence uniqueness and variation test (blue and yellow panels in Figure 1). Different from the original software, adenosine immediately following left PCR primer will not result in the drop of the probe set. Also users are allowed to specify the minimum Tm of hybridizing sequences. Since probes in which the PCR primer sequence was followed by an adenosine had a >2-fold lower signal strength [2], the final score (See Additional file 2: Score calculation for probe sets) of this type of probe sets will be adjusted by a factor of 0.5.

For MS-MLPA (See Additional file 3: Diagram of MS-MLPA), the probe screening inherits all criteria in genomic MLPA probe design, with an additional restriction enzyme filtering process (orange panel in Figure 1) [3]. Firstly, one and only one methylation-sensitive restriction site should be present in the LHS (left hybridizing sequence) and RHS (right hybridizing sequence), otherwise it will be hard to distinguish which site is digested. Secondly, the union of stuffer/tag/primer sequences to the hybridizing sequences may introduce new recognition sites for the methylation-sensitive restriction enzyme being used. Some methylation-sensitive restriction enzymes also digest single-stranded DNA although at a much lower rate. However for methylation frequency study, this might cause extra error to the result. Therefore, LPO (left probe oligo) and RPO (right probe oligo) should not contain extra recognition sites. Thirdly, at least 4 nt on either side of the restriction site should still hybridize to the target sequence, because the enzyme may be less efficient if the restriction site is too close to the end of the double-stranded region of the probe-target hybrid [4]. Lastly, in MS-MLPA, a mutation or SNP within the recognition site of the restriction enzyme could influence the digestion and might yield false results. Therefore, probes containing SNP(s) within the restriction site should also be dropped.

### Output

A link will be sent via email to the user upon completion of the analysis. The result page displays all probe sets passing genomic MLPA probe design criteria sorted by scores (See Additional file 4: MAPD result page). A MS-MLPA filter is incorporated in the result page, and can be enabled by clicking the 'Enable MS-MLPA Filter' link. The restriction enzyme recognition site and enzyme name will be displayed for probe sets that pass the restriction enzyme test criteria (See Additional file 5: MS-MLPA filter). The methylation-sensitive restriction enzymes used by MAPD are type II enzymes that cleave DNA within their recognition sequences. In addition, the recognition sequences must not contain multiple CpG dinucleutides (See Additional file 6: Methylation-sensitive restriction enzymes).

### Stuffer sequences

The stuffer sequences based on Lambda phage in the original software was suitable for human genomic MLPA only. With the software expanded to MS-MLPA and other genomes, new stuffer sequences need to be designed with the consideration of multiple genomes and restriction enzyme recognition sites. A set of new stuffer sequences has been created based on phage JS98, KVP40, N4, Phi1, T5 (See Additional file 7: Stuffer sequences). All stuffer sequences have been verified to meet following criteria: 1) The union of stuffer sequence and default PCR primer (used by the commercial MRC-Holland MLPA kits) should be free of secondary structures; 2) The union of stuffer sequence and default PCR primer must not have significant homolog to any of the target genomes (human, mouse, rat); 3) The union of stuffer sequence and default PCR primer must not contain restriction sites for any of the methylation-sensitive restriction enzymes used by MAPD; 4) No interactions should occur between different (stuffer sequence + default PCR prime) union sequences.

### Physical-chemical property verification tool

MLPA has been successfully applied in SNP genotyping, copy number analysis in segmentally duplicated regions [2,5]. Both applications put the variation at the 3' end of the LPO. The choices of possible probes are limited, and manual probe selection is usually sufficient for these applications. The uniqueness and variation test that applies to genomic MLPA probe design doesn't apply in this situation. However, physical-chemical property test can still be useful by estimating the secondary structure and melting temperature of probes, and therefore gives an idea if the probes are likely to succeed. The physical-chemical property verification tool offers the convenience for multiple probe verification (See Additional file 8: Physical-chemical property verification tool).

### Brief discussion

Most MLPA labs use commercial probes from MRC-Holland, or design own probes according to the recommendations of MRC- Holland synthetic probe design protocol http://www.mlpa.com. MAPD mostly follows MRC- Holland probe design guidelines, with a major difference in Tm calculation. MRC-Holland recommends RAW program to determine Tm, while MAPD uses UNAFold. A brief comparison of Tm calculated by RAW and UNAFold is available at http://bioinform.arcan.stonybrook.edu/mlpa2/help_Tm.html. Users are recommended to verify MAPD results with RAW. MAPD provides a rich set of stuffer sequences that have been verified not to interfere with MLPA. With future release of new assemblies of supported genomes, stuffer sequences should be verified using new assemblies.

## Availability and requirements

MAPD is available at URL http://bioinform.arcan.stonybrook.edu/mlpa2/cgi-bin/mlpa.cgi. The physical-chemical property verification tool is available at http://bioinform.arcan.stonybrook.edu/mlpa2/cgi-bin/probeCheck.cgi. Browsers should have JavaScript enabled. MAPD software itself is free for all users. However, since the software utilizes the UCSC genome browser and UNAFold, commercial users need to obtain a licence for those programs.

## Abbreviations

MLPA: multiplex ligation-dependent probe amplification; MS-MLPA: methylation-specific MLPA; CNV: copy number variation; UCSC: University of California at Santa Cruz; SNP: single nucleotide polymorphism; LPO: left probe oligo; RPO: right probe oligo; LHS: left hybridizing sequence; RHS: right hybridizing sequence; Tm: melting temperature; nt: nucleotide.

## Competing interests

The author declares that they have no competing interests.

## Authors' contributions

JZ designed, implemented the software and web interface, and prepared the manuscript.

## Supplementary Material

Additional file 1 **MAPD input page**. If the user chooses electrophoresis-based stuffer protocol, a stuffer sequence select option will be displayed. The browser should have Javascript enabled. Click here for file

Additional file 2 **Score calculation for probe sets**. The final score for each probe set is determined by the scores of Tm, ΔG, GC content and ligation site. Since each individual score falls in the range [0, 1], the final score should also fall in range [0, 1], with 1 being the best score. Since probes in which the left PCR primer sequence was followed by an adenosine had a 2-3~ fold lower signal strength, the final score of this type of probe sets will be adjusted by a factor of 0.5. Probe sets with a final score > 0 are processed for further tests. Click here for file

Additional file 3 **Diagram of MS-MLPA**. The principle is similar to genomic MLPA except that the sequence detected by MS-MLPA probe contains a recognition sequence for specified methylation-sensitive restriction enzyme. Only methylated target will be amplified. Unmethylated target will be digested therefore PCR amplification is prevented. Click here for file

Additional file 4 **MAPD result page**. All probe sets passing genomic MLPA probe design criteria are sorted by their scores. The "View failed probe sets" link displays failed probe sets and at which step they fail. Click here for file

Additional file 5 **MS-MLPA filter**. Users should select the methylation-sensitive restriction enzyme, click "Apply Filter" button. The restriction enzyme recognition site and enzyme name will be display for probe sets that are suitable for MS-MLPA. The browser should have Javascript enabled. Click here for file

Additional file 6 **Methylation-sensitive restriction enzymes**. The methylation-sensitive restriction enzymes used by MAPD are type II enzymes that cleave DNA within their recognition sequences. In addition, the recognition sequences must not contain multiple CpG dinucleutides. Click here for file

Additional file 7 **Stuffer sequences**. The stuffer sequences (used in electrophoresis-based MPLA) are created based on phage JS98, KVP40, N4, Phi1, T5 genome with minor modifications. Click here for file

Additional file 8 **Physical-chemical property verification tool**. Screenshot of data input page of the physical-chemical property verification tool. Click here for file
