# Supplementary figures and images for: MAPD: a probe design suite for multiplex ligation-dependent probe amplification assays
Source: BMC Res Notes. 2010 May 21;3:137. doi: 10.1186/1756-0500-3-137 (PMC2893534; doi:10.1186/1756-0500-3-137)

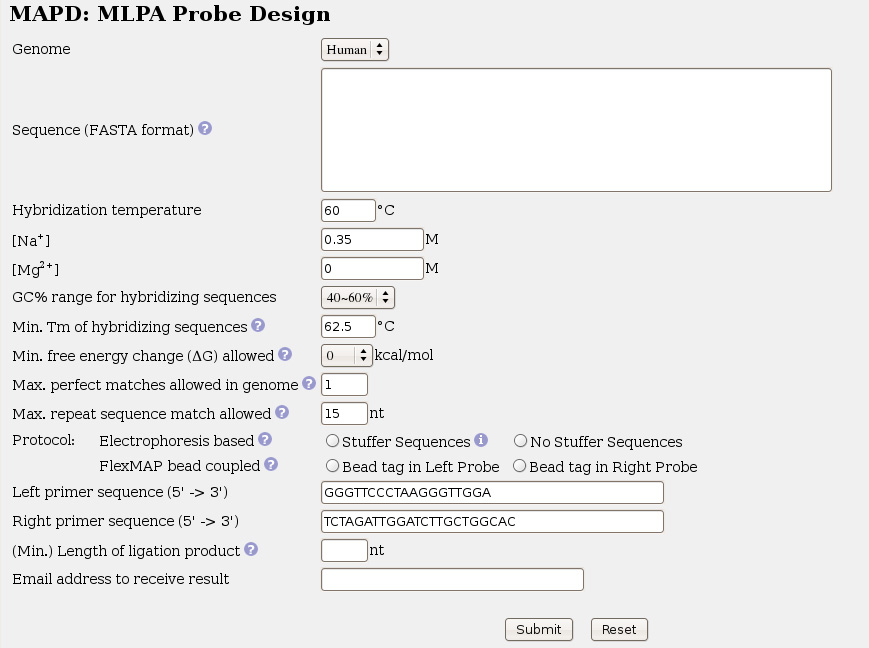

Supplement: Additional file 1 — MAPD input page. If the user chooses electrophoresis-based stuffer protocol, a stuffer sequence select option will be displayed. The browser should have Javascript enabled. [file 1756-0500-3-137-S1.JPEG]

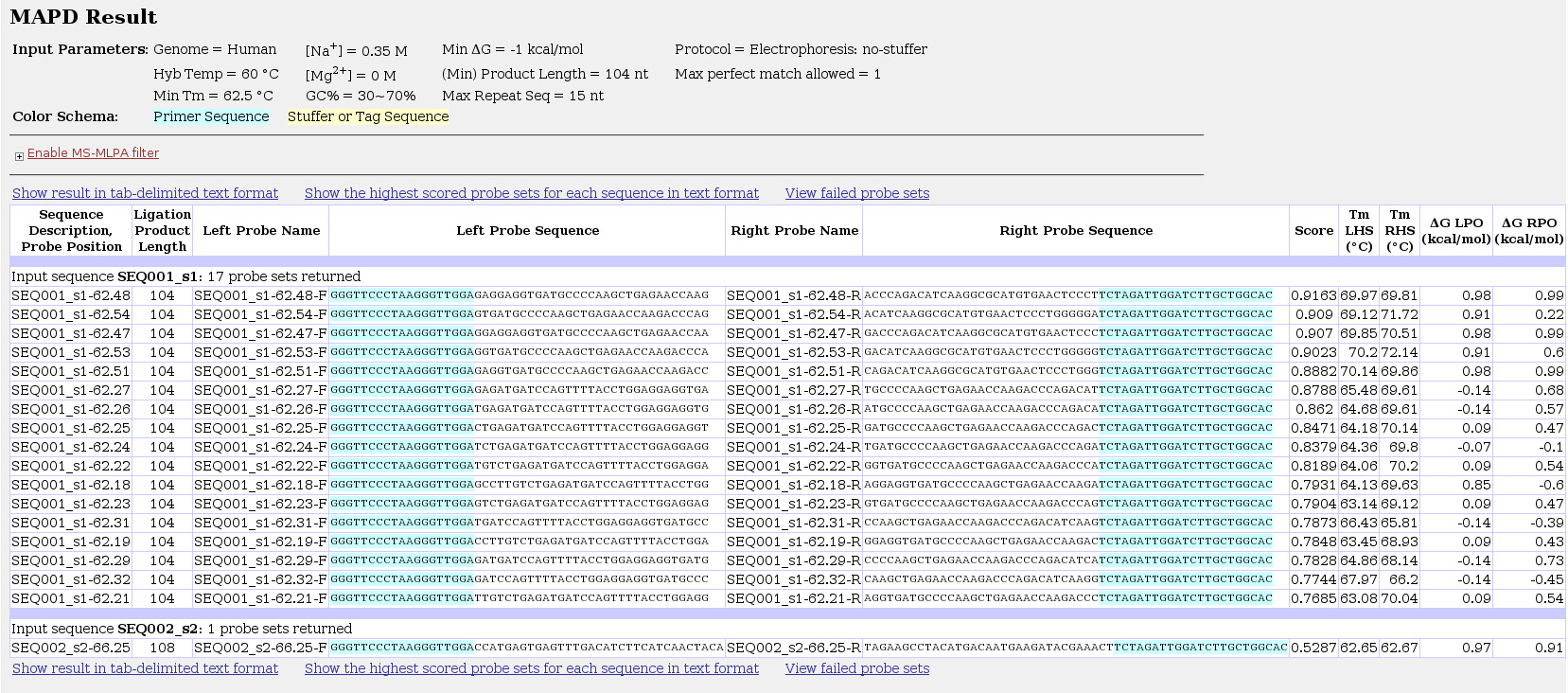

Supplement: Additional file 4 — MAPD result page. All probe sets passing genomic MLPA probe design criteria are sorted by their scores. The "View failed probe sets" link displays failed probe sets and at which step they fail. [file 1756-0500-3-137-S4.JPEG]

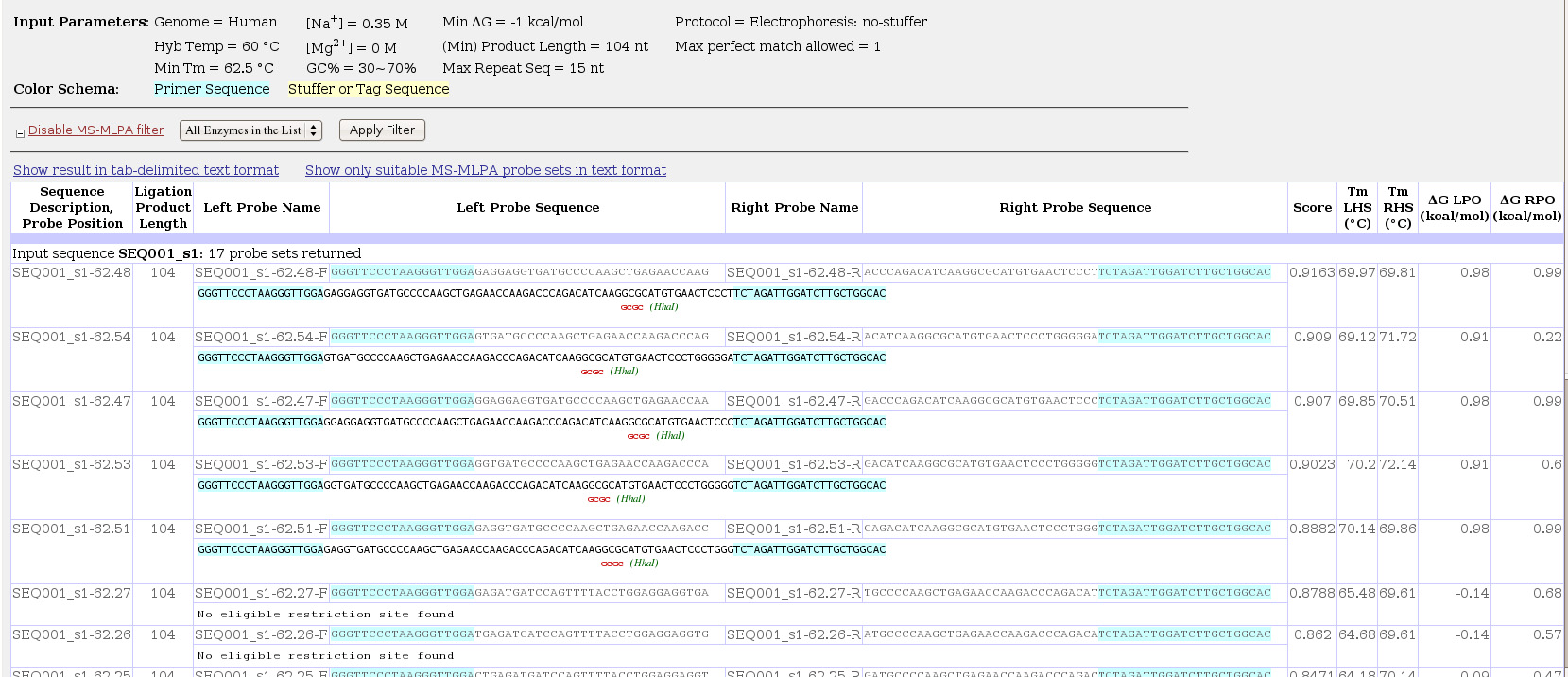

Supplement: Additional file 5 — MS-MLPA filter. Users should select the methylation-sensitive restriction enzyme, click "Apply Filter" button. The restriction enzyme recognition site and enzyme name will be display for probe sets that are suitable for MS-MLPA. The browser should have Javascript enabled. [file 1756-0500-3-137-S5.JPEG]

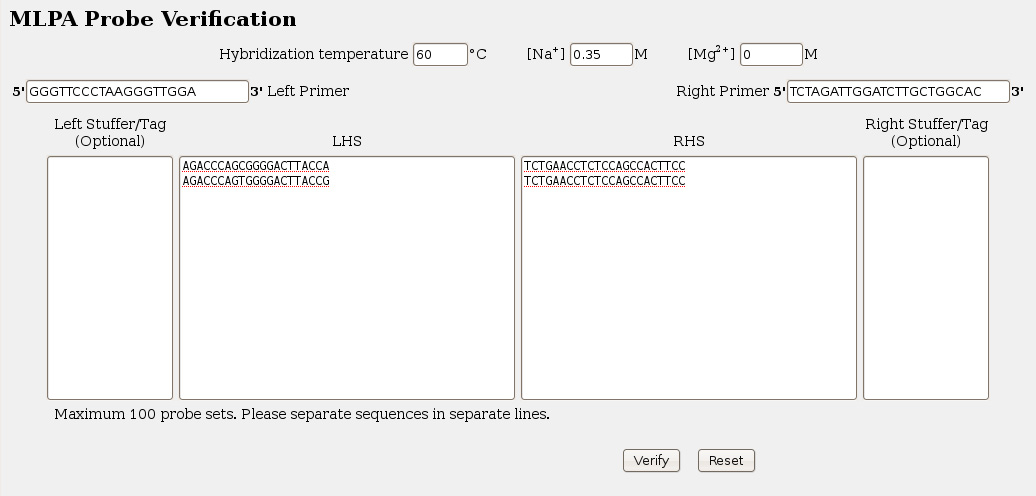

Supplement: Additional file 8 — Physical-chemical property verification tool. Screenshot of data input page of the physical-chemical property verification tool. [file 1756-0500-3-137-S8.JPEG]
