# Supplementary material for: MAPD: a probe design suite for multiplex ligation-dependent probe amplification assays
Source: BMC Res Notes. 2010 May 21;3:137. doi: 10.1186/1756-0500-3-137 (PMC2893534; doi:10.1186/1756-0500-3-137)
Supplement: Additional file 2 — Score calculation for probe sets. The final score for each probe set is determined by the scores of Tm, ΔG, GC content and ligation site. Since each individual score falls in the range [0, 1], the final score should also fall in range [0, 1], with 1 being the best score. Since probes in which the left PCR primer sequence was followed by an adenosine had a 2-3~ fold lower signal strength, the final score of this type of probe sets will be adjusted by a factor of 0.5. Probe sets with a final score > 0 are processed for further tests. [file 1756-0500-3-137-S2.PDF]

Tm score (S_{Tm}) for LHS and RHS:
if $Tm < T_{min}$, then set $S_{Tm} = 0$
if $Tm > T_{min} + 10$, then set $S_{Tm} = 1$
if $T_{min} <= Tm <= T_{min} + 10$, then $S_{Tm} = (Tm - T_{min}) / 10$
where Tm *is melting temperature of LHS or RHS calculated from UNAFold,* T_{min} *is the minimum hybridization temperature specified by the user.*

ΔG score ($S_{\Delta G}$) for LPO and RPO:
$S_{\Delta G} = \Delta G - \Delta G_{min}$
if $S_{\Delta G} > 1$, then set $S_{\Delta G} = 1$
if $S_{\Delta G} < 0$, then set $S_{\Delta G} = 0$
where ΔG_{min} *is the minimum ΔG specified by user.*

GC content score (S_{GC}) for LHS and RHS:
if $GC\% < GC\%_{min}$ or $GC\% > GC\%_{max}$, then set $S_{GC} = 0$
if $GC\%_{min} \leq GC\% \leq 50$, then $S_{GC} = (GC\% - GC\%_{min}) / (50 - GC\%_{min})$
if $50 < GC\% \leq GC\%_{max}$, then $S_{GC} = (GC\% - GC\%_{max}) / (50 - GC\%_{max})$
where GC% *is the GC content of LHS or RHS in percentage,* $GC\%_{min}$ *and* $GC\%_{max}$ *are the minimum and maximum GC content percentage specified by the user.*

Ligation Site score (S_{lig}) for LHS and RHS:
S_{lig} is based on the immediate 4 nucleotides next to the ligation site
if all the 4 nucleotides next to ligation site are G or C, $S_{lig} = 0.25$
else if all the 3 nucleotides next to ligation site are G or C, $S_{lig} = 0.5$
else if all the 2 nucleotides next to ligation site are all G or C, $S_{lig} = 0.75$
else $S_{lig} = 1$

The final score for a pair of LPO and RPO is calculated as:
$S = (S_{Tm}(LHS) * S_{Tm}(RHS) * S_{\Delta G}(LPO) * S_{\Delta G}(RPO) * S_{GC}(LHS) * S_{GC}(RHS) * S_{lig}(LHS) * S_{lig}(RHS))^{1/8}$
