# Supplementary material for: MAPD: a probe design suite for multiplex ligation-dependent probe amplification assays
Source: BMC Res Notes. 2010 May 21;3:137. doi: 10.1186/1756-0500-3-137 (PMC2893534; doi:10.1186/1756-0500-3-137)
Supplement: Additional file 3 — Diagram of MS-MLPA. The principle is similar to genomic MLPA except that the sequence detected by MS-MLPA probe contains a recognition sequence for specified methylation-sensitive restriction enzyme. Only methylated target will be amplified. Unmethylated target will be digested therefore PCR amplification is prevented. [file 1756-0500-3-137-S3.PDF]

MS-MLPA

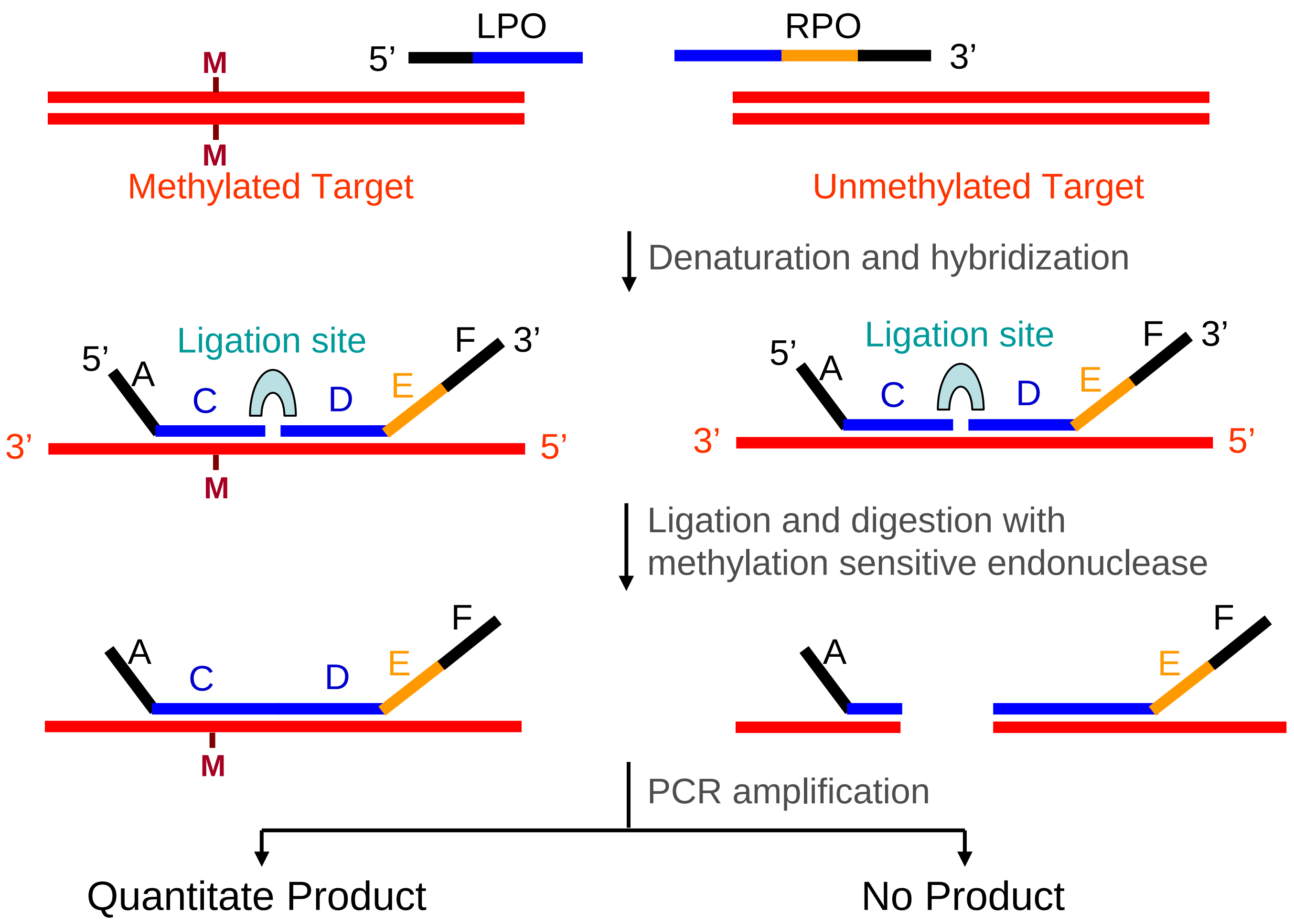

A: Left primer
C: Left hybridizing sequence (LHS)
D: Right hybridizing sequence (RHS), 5’ phosphorylated
E: Stuffer/Tag sequence
F: Right primer

LPO: Left probe oligo (A + C)
RPO: Right probe oligo (D + E + F)
