# Supplementary material for: MAPD: a probe design suite for multiplex ligation-dependent probe amplification assays
Source: BMC Res Notes. 2010 May 21;3:137. doi: 10.1186/1756-0500-3-137 (PMC2893534; doi:10.1186/1756-0500-3-137)
Supplement: Additional file 6 — Methylation-sensitive restriction enzymes. The methylation-sensitive restriction enzymes used by MAPD are type II enzymes that cleave DNA within their recognition sequences. In addition, the recognition sequences must not contain multiple CpG dinucleutides. [file 1756-0500-3-137-S6.PDF]

Methylation-Sensitive Restriction Enzymes

AatII	GACGTC
AciI	CCGC,GCGG
AclI	AACGTT
AfeI	AGCGCT
AgeI	ACCGGT
AvaI	CTCGAG,CTCGGG,CCCGAG,CCCGGG
BceAI	ACGGC,GCCGT
BmgBI	CACGTC,GACGTG
BsaAI	TACGTA,TACGTG,CACGTA,CACGTG
BsaHI	GACGTC,GACGCC,GGCGTC,GGCGCC
BsmBI	CGTCTC,GAGACG
BspDI	ATCGAT
BsrFI	ACCGGT,ACCGGC,GCCGGT,GCCGGC
BstBI	TTCGAA
ClaI	ATCGAT
FauI	CCCGC,GCGGG
FseI	GGCCGGCC
FspI	TGCGCA
HaeII	AGCGCT,AGCGCC,GGCGCT,GGCGCC
HgaI	GACGC,GCGTC
HhaI	GCGC
HpaII	CCGG
HpyCH4IV	ACGT
KasI	GGCGCC
NaeI	GCCGGC
NarI	GGCGCC
NgoMIV	GCCGGC
PmlI	CACGTG
SalI	GTCGAC
SfoI	GGCGCC
SmaI	CCCGGG
SnaBI	TACGTA
