# Supplementary material for: MAPD: a probe design suite for multiplex ligation-dependent probe amplification assays
Source: BMC Res Notes. 2010 May 21;3:137. doi: 10.1186/1756-0500-3-137 (PMC2893534; doi:10.1186/1756-0500-3-137)
Supplement: Additional file 7 — Stuffer sequences. The stuffer sequences (used in electrophoresis-based MPLA) are created based on phage JS98, KVP40, N4, Phi1, T5 genome with minor modifications. [file 1756-0500-3-137-S7.PDF]

Stuffer Sequences

JS98_v1

Increment	Left Stuffer Sequence	Right Stuffer Sequence
4	tc	tt
8	tttg	caat
12	tgatat	gaattc
16	taacacca	atcaaatt
20	tacgacacat	agtagtggtg
24	ttgatgtcaaag	ttcttagcatca
28	ttggatatggttac	gaatctcaccaaac
32	taatcgctctttagtc	tgtgaatcaaaacgca
36	tattgctcatatcacgaa	accaaataggttgtatcc
40	tgtgatttggatttgttcgt	atatccttaagaactataaa
44	tggcattatcagcatcagtaca	ataagcaaattcggtttcggtc
48	ttaggaataatacgcactttaact	tctttaattccacctactaaattg
52	tgctatgatgatgtctaaaaataggt	ctgtctaaacctacttccaataacca
56	ttcactccacgagtctgttgtacttcta	catagtaccgattgaagcgttagaacca
60	tggatacgggccttaactgcgataacagaa	tgtcttatcagtagctcccgatttaataag
64	gaattattttgtactcgatgtcgtatgggtct	cttttgatagtttcgatttcaacaatctcaaa
68	gaatcataggaaacctcgtttaaattcaacctta	tcaatatcatcagtctcgtgacggatttcagcat
72	gactcaggtttgccatctgaatggtacgatagagtt	acgattatattcaggcttatctttagctaaacgaac
76	gcatttctttggaataggtccctggattaacagagttc	accaatgcaggccattctttagtaaaccataaccgatt
80	gtgcattgcattgagcgaaaataatttagacttaacagca	ataagttcttgccaatggtttaattctctgttaagaagtt
84	gagcagctcgtccagcataaatcttagccaaaccaatatctt	cagtaaagaacacacgattcaggtttttcttacgaccagttt
88	gatgtgagcagtgccatcgggtaatgtaagcataatttcctgct	taacagaacccattgacttacggattatatctaacgatttctca
92	gcaatattaagcataggaatacgagtaggaacttcgtcacgaccat	taaacaatttagatttggtcaggtcggcagtcattttagaagtaga
96	gagtaaacaatattacttggataaccacggtcaaggatataaacgaaa	ttagggtctaaaaacttatacagagtttcaactaaagccttatcgtta
100	gtgtgtaagcaaattgtgatatttcaatccaatttttcaaggttcttaa	gaagtttttcagggtcgcctactttgaaatcgttaacatgggtaatgcct
104	gaatttctttacctaattcgttagttggtactttaatagccagattctcggt	gttccaactcaggtaaaaattaatgattaattctacagtagtttatcgttct
108	gttagtatcacggaatttgtcttcgcctactttattgatgattgcatcagcctt	aatccagagaataagaaggctggttggtaaagctgaatttttttataaaggtcga
112	gatgttacctcgatgtcgaagttagcaatacgaatcttggaagaatcatacttgat	acaattttaccatcgtctacagtgattgcaaatgtatcaatactcagaccagagga
116	gcaaatcctttattactcaggtcactcatggagtcgctagggtcatcatctacgataa	tgctgtagtattttgatatggtccatgatatcagtcgtgttttcttcacacatccat
120	gttggtactcgtaaaggacttgactgggataaccgtagttattatactaacgaagggttt	tcattcaggagttcttctttcatagcaatcatttccaagtttttatcgtaattagagagt
124	gtcccaagttttgataacttcgtcttcattagttccatacgggatgtaaatctggataagga	caacaagacgattatggattaatttaccgtattaaagccaatactattcgctttaaagcatt
128	ccttacctaatctactgaactctttggttttatcaggaacgaaattaggatggatattgttttc	cttttcggtatagactaatgcagttttcatgcgacctcaaattcattatcatcgttagtccaaa
132	catatttttctaggtgttcgtattttaaatcatacacatcagatagtatattattatacgaacgag	aattggtgtatgggccatgatagggctgttcaagtcgttaatcaagtcttctaatgtagcaggctc
136	caatttggtctacccatttattgatattggaaactatcacctcatatggggttaatggaggggtattg	ataatttttcatagaatttagcgaaaggccatgcgatacacattataatgcccactatccaaaacata
140	cattagcacgagacaacatacctgttttacgaagagttttcatgactcggtcttctaaggtaaacgaacc	aattgagattatcaactctaacggaagtatttcatctactaactatcttgaaatccgtatattagatact
144	cagtgaaaaaatatctttagcgttcatgataacctccagtagttgataggtctatagtatcatgtctaatgg	caagagcagtctcctgtagtctccttcttcgcaatccgttttataaaccttctcagcgaatttgtgtttaga
148	cagcttcaataatttcgtattcttcagcacgatcagctttaaaggtatcttccagagcgatcaagttagtagtg	taaaacaaaccctagactcaacagaacaggagtacttaatacaaagaaatgctctaatacaccagctcctgcaa
152	cagagttcagataagagactaaaacttttagctcgtctttagtaaataccacttcattttcaccatcgtgtctttg	cagccaaccaatataagaatctacttcagtccctcgactaagtacgaaatcattgaatttcatcatctaccttcat
156	cgattgcttcggtatgttcatccaaaagctcgtgaataagctcaaacaatctacttttctgactaatacttaaactca	gtcggatttgtttgctagtgaagatgttgggcttaaacatccaatcataaggatacaagccactgtacggaaaatacc
160	cttggtttaaacaaaaggtcatttgcttgtagatttaattctatcatacagtttactaaagcaccgtaatcaagattagg	gttcattactgaaagtaggctctacgctataggcagacatactaactgtcaatagtaaagtggctaatactttttttcatg
164	cacggaatttgcatgcagtgttaccaataaaactacgaacagaatcagtgtacaaacggtacttaatactttctgtatggtt	ttacctttagtaaaatcaaccaaatccacttgtacgccctgtttaccgaggcttgctacaatagcatcaacatattcttggt
168	cacgatattcagccattaagtgttggtcagccaattcagatactaaggtaaggttgatacgagtcatttttaatctcctgtagt	atcaagacctttccatgctgatttatgcttggcaaattcaagaccagcgaagttgactgctttagccaatttattatcaatagt
172	ctgaacatttaaccaggtcagcagatttcaattcaccaaatgcttgacgaaggtcaccgtcacgagtgttaaccatcaggcattta	gttcctggctcttctttcgtgggtgatttgagtacttcacatgttatatcagcagctgatttgaatatttccgttaacagtcctat
176	cagaccaaagcgttgtcaccacaatcattgagcttaaaatggtcatagtacatagctatgtgtctagtctcgtcaccagagaaccccta	taacgaaatacgagtaggtttttttgccaaaatattcttcataagaaccatctaaacgacctgctttagaccgacaatacgaatcgtac
180	cacagaaacatcccaaggtattattcgtcttgccacaacacaagaagtaatggatgggttggatgcttatagtgcagtgactccagcaac	atttacattccttaataaaggtttcaaggtcattacggttggtttgttttacatactcattataattagccatctctaaagcatatctct
184	ctaattctttggcagctttttgttgttcaggtgttaatgaattatataactcttgcatttctttatcttcgatattcattatgttctcctca	atgagtcgctcgcttgaccgtccagctacagggccttaatttaaattacaaatccagcaggagcattatcgtctttagccatgtgctgaatt
188	caagtagtattcttcttcgtcatcgttcagcaaggcttctaagtcttggataccgaggactttatctaaaaattcaggagtcaattggcctgat	gtctgggttttatagttatcgccatcattttcccaggtttcaatttcaattgcataacctttagggataatttgtccgaatttaatattcattg
192	ctttggtaagaaggtcacaatcagctttaacacgctgtgcatggtcctgagtatcaaggatacgagatttcaggataacgatttcagactgcagtt	catgaccaattatcagatagcgttccagtaggtactatttgtatgtgaggtggacatgaaggtaatatcccaggcaaatggcgagcttgtgtaggt
196	cagccaggaagaatttaaggattacgatttcgttggttctcgaatgaatatccttattgatatgcttgcttataatactctttatatacagcaatttg	atattatctaatcaaataggtcgagaagttaaaggtaaccctggtgcaatatcaatggacgaggcttctaagttattccaagaagaccttaaaaaagt
200	cctgtttaaattgctttataatgaagatgtcgaaattgatatcgagtccaagaactctatcgaatatgatatcatcgtagaatccgacaatatcagtgaa	taagaaaaactcttactaaggtttataccaaggttatagtgggtaattggagcaaatgggctaatcaactcagagaatatgtatatgcgaggccataatg

KVP40_v1

Increment	Left Stuffer Sequence	Right Stuffer Sequence
4	tg	at
8	ttgc	gttg
12	tcattg	acatga
16	tgaacagt	tttgtatt
20	tcaacgccct	gagtgaatca
24	taccatctagta	cgattgaaaatc
28	tgattcgtgaggat	aatctcattgagtt
32	tatgcgtagtgtacct	agcagcttgtgtcact
36	tcctgtaattttcgttga	attcctatacaattgctg
40	ttcctatacaattgctgtgc	tatagtaggtggacaagtca
44	ttacaccaaagacggtctttac	tgcttgatgatgaaagacggta
48	tggtttacaaatacccactgatca	atattttccgaattacagaaaata
52	ttcgatggtgatatagcaactgctga	cgaaatctacacgattaagtgcagtc
56	tctattcactttccacatggcatccctt	ctgtttgaagcaatcgggattatcatta
60	tagtgttgtctcctattattatctcatcga	aacgaattaacaaacaagagagaataatta
64	gtattgacaataacacatcaatacccgataat	aaacacatcaagacgagatacgaactacttaa
68	gtttggtcaagttattgttgagctaggcactgca	gaacaactgggttcgtgatttgaaacacaaatat
72	gtgaggtagtatgtacgcaatacaagcaactagtca	gtcgagctatctgaagcaggttatcctcttcttgat
76	gttgaaagatgacggtgtgaaatcaatgattgttcgtc	aatacatcccgatgatcaacaagtacatcaatgattac
80	gaggaaatacacatgaacttagacaatcacttatcaatgc	cagagataaagcactgggtcatcattgacagcttcactgg
84	gcatcgccaataacaaaacaaacactctgtaccagtcatgtc	atattcttcagaagcgtggcgtttactttctggtacacttca
88	gcaatcgtagcactagcagcatcagtagcaatgactggttgtac	cttgtcaccatggggtaaagttcaagtatcaactacagtcaaca
92	gttcgtatcgtgtctcgcccgatggagaagttcttcaatctgaacg	agaatccctttacgatggatcttgacttgacgactactgttcagct
96	gagttgtgtgtaatcgtaatggtcgtcaatgtcatacaagtccatgta	atcacacgattttcccgacacgattttctgtgcttgaacaccttttcg
100	gagactggcgacataccattgtgtaaatgtgtcgagggtcaaggtgtgaa	gtctgtagatcatgtgatttgaacccattcgttcccatgtacctagattc
104	gtcgtaagcgtgttgcagttcatgtaatgcagtgctatacaactgtggtttc	tacacatacaagtacgagggttaatctcatgggcgattttctattggcaatg
108	gattcgtgaagttgttgttgaagaagtcgcacccgtgtacactaacattaaagt	gaatttaaaaagcagtttatgcaactaaaaggaaagattctcggctgtcattgt
112	gacaaagacacgataccagacggggaatttttagaatgaagtttagtgaatatcaa	ctatttttgactatggcgagggtcctgaagttgatcgaacatatctagtcggggca
116	gagagaaatatattatgactactatcgtaaacttcactgctgcaatcgaactaactac	caactacatacgaagaagagttccaacgaatgggagttcagcgtgtgtgggtatctga
120	gttgaaaacggtctaactgatgatgaaaagaaagctattcttgaagatcgtttcactgac	tgtagcacttatcagaagtggttattttggtgatgactttcatatcagctatgactcatc
124	gatcaacaaataggagtgatgggccctaactcatcaattcatcaactttcgtgagatctata	ttccttttgctcttgtagcttcaaacgactgaagatcttagtttggtctttcggatgttgtg
128	ctcaaaaggttcaaatgcagtcacatgtttgccaccgattacttctcgaccccattgtgcaatc	gtatctcacgatgaagcacttgaagcgattcacgaggtacttaacgaaatgtgagaaatatatg
132	cagcaactgtaactgcgagtgaaatattcgagctaccatcaaacgaaactgatccactaacatcac	gaaaagttacaaactatccaaactgctccgaaatacaagttgctgtttgatttggtaagttcaaac
136	caaaggtgttgaagagaaccgtattcgtcatcttgactacagcgtatcaattaaccgattgatgatgc	gaatgcgtggattacatctaagttcgacaaaagcatacagaatcgtgtgttgaagagtcaaaacgatg
140	ctgttaaatgatggtcttcaatgcgacccaaatcgtgtcgtagttgtgaccgaacaaattcaaaataacg	ttcagcccgtgagtaaagagggacgactactcgatactcattatactgacattgaattagtcgaaatcat
144	cattggtaaaacagatatactcgatcacaaatactcatgggcgtttcgtggcaatgagattcttaaatggat	caatgcccctttgaggaatattatgaacatcaatctgcttattcgcaatgaagtgaagaacaacacgagtcg
148	caattacatcactactatcatggtagaagttgatgacttagatgaacgcattaccaataagatcgttgagttgt	caagctatgtcgcttctcgattcaatctaatagtaggataactcatgtttgtactatcactcttaatgcttatt
152	ctcgaagaagttcacaaaaagattgttattccagatgtatccgatttagataaagtattaatgtcgttagacgaag	gtcgatttgaagaatacaagtcgtatttcccagatgtgtatggagagacaaaatgatttaccgtttctggaaaaca
156	cgaagcaacttcttccaccatgggcgtttaagctcttgtacttcgttctgaagcgtgtctactttttcattaagctct	acaagtttctagatgtcatgaagctagttgaagatgaccccgaagaagcgtattcgattgaagtcgatgccaacggaa
160	caaaaagtggttgcatggcgaatggtaatctcctattatagctacatcgaaacgagatacacaatcttaaacgacttaac	tctttgaaataatgcttgacgagtttcaccaaaaagccgataatagctacatcaaaacgaggagaacattatgaactaca
164	cagcgaccaaattcgtaggctcaatcgtagctgtatggcttggtattatatttctcgtagccatgaacatcactgggtattc	aatgaaaaagcgttatatctaccgttactatgagaaacttgattatgaaagcaacgcaaaaacagatcgaactatgtaactt
168	cagatgtaacttttgctaggtatttgttaacaatccctgcgaatgtttgagttgtcgagaactcggaattttgtgcaaatcgag	gtaacaagcacaactcacatcgaacttgaaatcacaactgaagtacttggttggttcaataatgaattcaatcaagctgacggt
172	cctaatcgcagccccattttggtctaacaacttagcaggagcattgatgtttactgcatagtctcggcgatacttgtacatccctc	ttcatttccattctcacacggtaattactgtagcaacacacaaggctattactttgtcaatctatgggcagagaatcttaaacatc
176	caaaacgagcatcagattctgtttcagtgtagtaacgaccatcatgcgtgtgtgaatcgttcgctacagtcgctgtaacggtcacagt	tgcatatcacacagtcgaagaagttcacggtgtgtttggtacaattcgcaagccaccaccgaaactaatcgaaggtagggcatatatg
180	ctcaatgaaaagcttgaatccctcaagtcagaacaattcggttgggttgttgcattacctaaaaaagaaggtgatgacattgcaactgtt	taaagctttattcagcaaacgcaaatggaatgttgtaggtcgttctgaaaatctcgatttatggacagtgactactcgcagaccacaagt
184	caccacggttaatgcagttgtgaacaaaaaagagcgtgaaatgattaactactggatggcagatgctgacggtagtaactatctttacaaca	cgatccgtttgacgaaaattactgctatgcgttgaatgctgcaaaatacccacagcgtattgcaaaatcccaactcgtaaccgatgagcaat
188	catattgcatcacttgagaccgagagggattcgattcttgcatcatatgatgacaaacttaatgagatcgtgaccaaatatgaacccaagcaat	gtgattctagaactagggggtaatttcgataatgtatgattatgtaattcacacttcgattagtgacaaaagtcagctaaaccaactagtgatg
192	cttgttgcacaagctgaaagtgttacactcgatgagtggatagagatagtgcgacattttaaaggcaataagcgaattttctacgggattggtagc	agtgtgaatatgcttgtgaaatgtgcagttcatcaatctcacatagctatgacaagaaatacaatgacggtaagttggggattattgagaatgact
196	cttcattgaacaatctagccttcaagtctgataatttgctgtagcagtaacgacaagcttgattacattcattactagggaatacttgtagccagcga	catcatgaagaatggcaagttggctttccagttaagcaaaaccccgtctacatctatgatgatcgtctttttcattactacctcgaaaaaaattaaaa
200	ctatactaaagtctcacacaatcaagcataaaatgggagccatacaatgctaaatcttaatcaaaatatcgcaatcgcaactgacagctacaaagtttca	agcagcttgtgtcactgaacaactgggttcgtgatttgaaacacaaatatctgtgaggtagtatgtacgcaatacaagcaactagtcatttcgttcaatt

N4_v1

Increment	Left Stuffer Sequence	Right Stuffer Sequence
4	tt	tg
8	taaa	acct
12	ttggtc	acttct
16	taaaactg	atccaaca
20	tggttctcgt	gaaagctcag
24	ttctggattaac	ttggctatgtag
28	ttccattgatgtct	tatgaagcgactga
32	taccactggatactca	gagcatctgccaacca
36	ttcctctaacgaagactt	gaaattgtaaaacattgg
40	ttctaaggttggtctgagag	cttgggtattgctggtaaat
44	tactgatgaagagttatggcgt	tctgacccagtgtttaaatact
48	tacagattgatgagattggttcaa	accttctcgctaatgctgatgtat
52	tcatagtcttcaatctgaacaaagtc	aagattaaacgcaatcaagtaagagg
56	tccaagtgattgtaggtgctacaacaat	gtctttttgcagtacagcttcagtaatc
60	tcaaaacgatgactattgaagacattcgta	catttgtaaacgccttcattgtctatgctg
64	ggagaatatgcgatgtctactatcgaacatca	gatgcacctcgaaaagctgtacaacaagaacc
68	gaaagagactgatactaacgaaatgattgtttct	tatggtgagaactttgcttatgactatattgcag
72	gtgaatatctgaaaattgacattgctaataactatg	gtctggataaagaagactgggatgaccgtattgcat
76	gttagaagagggttacttagtagagtatcccgattcta	tgagtagcaccattcatcagttcacgggacagatgttc
80	gagggtaggtgctcttaatccttgtccgaagcctaatgag	tgagtagcaccattcatcagttcacgggacagatgttcca
84	gagactgtccacttcttagataagccatatgactgtgttcgt	accatacaggttaccgttaacgatggtagcaccacttgcatc
88	ggtattttcagcattcattactacatgatatgccttattaccga	atgcaccgaggaacatcttcttaacagtatcaagggtgctaggt
92	gaactcttattaatcatgcagtaactgctgaatctgctggtactta	taaagtcatcgttactgatgccaacggtactactatcgagagttct
96	gaagagttcttcggccttatgacgagtctcaatgttcatcaggttaat	atcagccagtactgggtcatcaattttacctaacaggtttacatattg
100	gtgactttagaagcatgtattcctgacccacttaataaatagtatggctc	ataccgttaatggtcagcctagtctctgtcacatctggactggttcacat
104	gcaactcacattattgcctatacccattatcatggttatatccccaataaga	tatttgtaaacttattgcagcaatcttaattagtattattggtattgcatgg
108	gtttgggtttatgtaatgtaccgtatttgcagcgactaatacttaatatagctt	tctctttctcgatgagtttaactctgctcgtaaagagattcaggctgctgctta
112	gtgctggtaacaaagcaactgaccgtgccattactaacaacctgtctacagctatg	ctgaatgattttgaaccagaccatcaagagaagactttttgttgttctcgtacatg
116	gtgtagcaacatcattcgttcagtttactcaggtctatagcaacatggtgtctttgaa	gttccgtaggtagtctggttccagctttggttaaattaccgaactggcgtgttgctgc
120	gatacagcacaaaccgatggtgagcatctcgaatggaatcctgattggtttgagagtcta	gtgatatttatatcaacaatcagcttattcaggatggccacagctttataggaatcgaaa
124	gtgattgtcttggtttaggtgcttcaacatattagggtcaacggattgttcataagcaacta	ttgacattcctgtcatatgggtagttattcgtaaccctggtgcacaagttccatttgggaaa
128	ctctgttttaacaacagtagagttctcggcattaagaatctcaacctgccattggacagcatca	ttgctcttagagaaggacaggaattactgtctactctaccaaaagcagaatttaaactagccta
132	cagaatttatgactcaccgagcacttaatcgtaatgcttccagtagtcgtgctattcctacccaac	cttcagccagaagaaggtttgcatctggtcagagttagcaccatcaatactagatttctgaccgtt
136	ctttggtttacgcattcatcctgatgctcaaccagagatttgtgaattggctactaagatgaaggaac	acctgatttgctacttctgcattttctgaaccagcaatgtagaaagtctgagaaccagtctcaatcat
140	cgagctttcagtacatcagacatacgctcatagatggcatcagtaacagaactaaccagcttacctgcaa	tggagttacaaatgcagatttatcctctgctactaagtaacgggaatactccataagagccatcaatgct
144	cttatcttccgtgactatggttctggtattcctaaagataagattcaagaggtctatggggtatatggtgca	tgcagagaagtcttcattgttctggttcgataaatccaaagtagctttagtaggtaagatggcctcacgaac
148	caactggattccacattacggtaagcagtgtacaggttagaaccaggaacaaaggaacctagtttaaagcctat	agcagatattaaagcccgtcttccaaatgctattgagtatattgggaaagctattaaagaatatagccctcacc
152	caatgttcaaaacctttgctaaattggggattactaaaggtatttccctatttcattatcaatatggtgttgaact	cagccattgctgtagggatgcctttggtatagcccaatggagcatttggattacgagataatccccagtaagactc
156	ctttgctgtacataattaattacctaatctaattcctgggttaagagctgggtcacgggttgggtcaaactgactgga	taataagaaaaaggttagtcctaaccgtcttattagtatgaatatccttgaaataccaaaagagcatcgtagctattc
160	cgaatggttgatgtagatactccccagtattacatcatgcaggaagatattaataactacacaggaagacttggtattct	aggtgctgttcatttggatgaatggactgctccgtatattgcttcggttcttacatccaaacaattcattaagtatgtca
164	cttcagcagctatatcccgttcagatacagattgaatacctgtatcctccccattagtcaatgcattatacccaattgcagc	tgaaagctatgggtgctgtcgctactgatgcttacgagtttcatactgacttgaatgagaatgaagagactattgtagctgt
168	catgagttctttgtatcgcttaataacttccccattaaaagcagggttagcagtttctttctcagtaaagagttcataacgagc	aaactgttgcacctaagaaaactacagtcactcgtagtcggaatgctgatgttattcgtagttatctgaatgacaaaggtatgt
172	ctacaaatgagtgaagtatctggttcaaattagaagggttatagatttcaatacgatattgggctaatgcagggtctaatccgtaa	gcatcatacatataagatgcagcttccatcagtgcttgaggattagattcatctaatgtgttaactaagtctgctaacttctgaat
176	cttcattctgcttcataacccgttcaccacgagctactaaagtattcttaataggagaaacaaccttacctgctacactaccagcagc	tgctttacccagtaaagatgcaccccttaatacaggaccagcacctaatagagaacctacccccttctgctaacccatcagaaataata
180	caccaatctggaagtacagctccattcctaagttctgacattctttcagaatctgacattgggctttacttacaaaatccagagcatctt	cattagttaggttcattgggtcaatgtaacccataccctcagtaccaaacatttcctcgtacttttcctgaatggttttcatctggtcac
184	catttcatgactaagactgacactcgtattgttgcctacaccaaccaacaggttgttaactataacaccttcattcgagccatgaatggatt	ctattaccactgcgagattcccaccattcctgataagcatcaaagtccctgttagcttgagcaaagccttctaccaaagcatccatagttgg
188	cacttggttcacgaaattctttctcgtaaacatctgagaagttagtccaactaacctgttcttcttcagtattggtactttcctcatcaggtct	acctaaatcaataacttcagtgtttgtagtggaaccagtggatacagtaccagtctgtacctgggtcattatagtcttacgaataatgattaag
192	cagtcccatgtattaccccaatgcgttgttattcttcaatgcagtaagaatgctatccagactggagttagtgaaaccattaggaggagacagacc	ttagctttgataataccaagattagcacgagcagtaattgcagctacctgagcagtaacagcattccaataagcctggtctttagtggtaaggaat
196	cagcttgttcttgtgtttgaatagggaacaaactaaatacaggaacttcttgtttctctttacgaatttccctgttccaaccaacacgaacaatacca	tggatggatattctgtgggctagactttgcttctctcgaagaccgtatatctgctttaacaaccaaagaccctaataaactgaaggtgtataccgatg
200	cagttcagaacctacatacataacacgagtagcaccgatgactttagtatcaatcatacgggaaccagtgatgatggtagtctgagtaggagtacggttc	taccatcatcaacaaccgaacgaacatagttatcaataaaacttacacgatttagtttggtacggaattggtagttaagaaccagttcattctggcgtgc

Phil_v1

Increment	Left Stuffer Sequence	Right Stuffer Sequence
4	tg	tt
8	taat	tctt
12	tcttag	acggaa
16	taccagta	ccataatc
20	ttgattgcag	tcggtaaatg
24	tatgcttctttt	gttcaatgcgtt
28	tcattagaacatca	aaagcatgtttttg
32	tcttctgtttccatat	tcctccgtttcggatg
36	taattcttctttagcacg	ctgtttgatctctttgat
40	ttcaatcaaaacgctggtat	attttagcacgatttcggta
44	tcaagaccatagaccttactcg	ctaactcgtgagttttaatcgt
48	tatatttggtctttccgtaatcct	ttctcaatgctcgcccctgaatgg
52	tcagaccagattgaaagcctttttta	agatcacctgaaaacgcaatattcag
56	tccagtacagacattattaatacctcta	ttcagttaatccgtaatactttaattgc
60	tgatagcatatactgaccatctcgcacaac	ttcaaatacatggatcttagtgaatttcga
64	gtttgccttgtactaatccacatagcagaaag	atccatgaaatttttcatcgaccagtgcgttg
68	gaccttttaaaatatcaatctcccatcgttgagg	gttaaagctgaacatcacagcaacatttttatga
72	gaaactaaattctcaataatcataaatcgccctcag	ttctttatagcctccaatgtgctcgttgtcaatgaa
76	gcaactttaaacggtaatctcagatatttcaaataatc	aacatcataccatttaaaaaattggtttgcatctttta
80	ggaatctcccccacatgccttgttatattcgatccatgac	aggtctaagcagatccagaatcaatcccattgtatcatcg
84	gaatgtatgcaaacgaccagtagacaaatagctatcctcacg	ctcaaccagttcaatagcaatccttttagctcgttctttgtg
88	gtgaattgtcggtatagaccaatacagaattattatgatagcga	atcacatacaggttattatcatcgtcaattacgcaaaagctaca
92	gaatatttttgataatataaccgtcaacattaattggtcggatatt	tagtacagcttccagtttagtcgcaaaaactttaattgctggtgtg
96	gaattgctcgaatctcttcgatctctttttcagtctctttaacaattt	ctggatacggtttcagtattaaaccaatgaggcaaattatgtggattt
100	ggtttgtatcgttcgcctactttaaatctctgaccacgctcttgcatacg	tctagaaactcatctttaccaccttcacggaatgcatagtaatgcccttc
104	gttaaaactcgtccagatcgtaacttagccatgatttacccccaattcatca	gttatcatgaatccttgacataatgggcgaccttcaacaatgataggaagtt
108	gactttatcaagattttcggttggcatctttgctagtcgatgtgttggatctgt	cttgaatttctctatagttaattctttagtagcaagatgaataccataagcatc
112	gttaataagagtttctcgctcactaggattatttttccacaattttgtaataggat	gttgatacctaagccaatgaaaataggttggtcttctacattacccatcccaacac
116	gttaaatccgtttgttaggccaaatacaagcattccagtacacatactcagagcaaca	taggaacagtaaaggccatagctaacatagcaacgccacaaatgacattaagcaccat
120	gattctcccaagtgtttttcgtttctatgtgtgcattatgctatagtcgtttatggttgt	cgaaaaattttcggggtatagaattacagtttggtcgtccactccaggtctagatcttcg
124	gtgccattatttataatgatggtttcaccgtcaataggcaaaattcctttttcggtggagtg	ttgttttaaatggaagaattgcagtacacaccagagattcatgcatatcatcggtgtatgtt
128	catccatgtgtaaaggtttagatagctttctagatgttcatcgacaataaaccgaactaccaac	agtttaaagtaaagccttttgttagttgtccatctggaatattgatctgccagttactagggtt
132	cttgattggtaaagtttctgtgtatccatctttcaacatggatttgtcaacaacgccacaatgata	ttatgtacataggggtgtttaatggctggattatcctacgataaagctaaaactacagggcacgga
136	cagacctttagtaagtctaaaatgcttgctcgtattgacaaagctgatgtatctgtgataggttctag	catataatttttctatgatgcgagagttcaaccttgcaaacgagtttgttaagcaaaagtttacatat
140	catgtcgatacatttttaaactcttctgtcgtgaaacccataaactcgctatctgttaatgaagatttga	tgtaaacatgataaccaatggcacacatacgaagaaatgaacgagatgatgaaacagggtattgaattta
144	ctatcttaataacgaagatgaagttacgctgacaaatcgaattgaatataccgatttccagaatccaagcca	aattttgtttgattcaatccctgatatcgggactaaagtcactactctgggaactaattatgaacttctaaa
148	cttgaatctaaaatgatcatgaccacgactccgaacgggcttaatcacttttatgatatctggcaatcggctat	ttaaatatctcatgatgtataacgaatgtccagtttatatcgagctaaatagtacgggtgtatctattgctaag
152	ctcaaaaatgtaattacggaagatgattgggatcgtgaaataaataatatcaaggtggttttccatcgggactctt	tggtaaatatttctctaaccagacagtgatgcgtgacattctgaaatataccgatgaccagatggacaccgagaag
156	ctttcgataagaaattaactgcaattgtcgaaatggttgctaagaaagaagacaaacaagcgaagattgaagaatcac	gctgatggaagaaggtaaactggctgaaatcgctttcggtatggcgacttccattgccgaaattcaggaaggctttaa
160	ctctgaaaactctgtaattgatcttctggctactgttgcttccgaagagatcaacaaagatattattcagaagttgatca	catggtatcccatgtctacgggcaacataaacagcaattcgagttcctcccgaaacgattccattaacttctttcgccca
164	cttccaatttggaatgaaaatttttctataatctggatcgtaatacttcatgaaagcattgtccataccgtaagcccctcca	gcttttctagagccgattaaagcacatctacgaagtggttttactccaaatagatcttgtaactggtcaaacataatatttc
168	cattgaacattttcggcttgtacttgctgcaacttctactgtttcatcttcgatctgttgatatagtttagcaacacaccagaa	agccactgctaaaaacctgctaaacatcgttactgatatggatcattgccaatttaagatcggcatgactggttcccctcgtga
172	caacatcttctgtaaacttaggttgacgaaacttgatttttacttgtcctaagtccagaatatacggttcaacttctggtatctgt	gaagaaaaagatattctgcttcgcatttctctagctctaatgcattgatccccgtacattcatgtatgatctctataacaacctct
176	ctttatcaggattatgaggcatttctaaatcatcgctttatagaattgggtgctacctttgttacaatgactctgcgtgattcgattc	cgaaggattgttgcaattctatgataataaggcgatgcatacaaaattaaacgggagagagatcattcagatttcgctatccactgct
180	caataccgtttgtgctgttagatacctgagaagacgactcacaaggcatcagagccgaaagcgtactattacgaatgccatatttttca	gaatatacttctgatagtgaccatattctatagagttttcctctacaaactgagttgtcgcttcaagataagtcaatccctcttcaacaa
184	cattgcttccttgatcttgttctctaagtctaagatcttttgaccgtcttgtaaagtcaattcataaaagatctcgtgatcgaacttagaaa	aatgttatctgctgcgaccttaatcaatactttctggatatcattggtagacataccttctgtcagaagtggttcaaccatatcaaacaatt
188	ctattacccaacacggaaattatattttttattgcatcgtcttcgttcctgcgaccactacatactccacatctatatccaccatgaaaaaaat	tcttgcaatagggaaatcacatgctggcgatgttcttccgattgagggacaccgaaaaagtttgttttgtagaaatactctaatcggtcttctc
192	caaagttggtagtggttttagtcagtgccattgtttgattctcctaagtggtatctcgtttcgatgggtgtattatgctataggtcggatctggtg	agcgagtaatgctttacaaaattctgggttattccgtgccatcgtaacaaatgccagatgtaccagtctgtaataatgctcactcattcgatgaat
196	cttatcggcttcttcaaccgacataataccttctgcgacaattggcatagggtgatcgtgattccaccaatggtcatgtatatcaatttcagttttgc	tgcttatctgctcttgtgtaggcaatatttctatgctcatttagtagcctcctttaattcttcggtaaactccttaaatgaatcattaagctgtttct
200	ctcactacaaagtgtttgttgatagagtcggtgcaaataaatttcattttgattctcctttgtttcgtttcgatgggagtatcttactacccccttttatt	ttccattctggtttaaatttggtgatcaaaacaggttcttcgatatcggcagtgcttataatatccttacccgttggattctcaatatataactcaaagc

T5_v1

Increment	Left Stuffer Sequence	Right Stuffer Sequence
4	tg	at
8	tgtt	tcat
12	taaaag	ttatca
16	ttagcaag	cgaataat
20	tatatcttta	aatagaccca
24	ttgttatagttt	ccttatgttatt
28	tgttgggcttgaat	taataaacggactt
32	tctagccttgagggat	ttttagatgttatctg
36	tatttatctgctggatgg	ttgatgaaatggtgaaag
40	tattgatatttctactttta	gttaacaccgaaagcgagta
44	taattaacctaatgtaaagttt	ctgtataaatatacagtaggat
48	taagtctgttgataacgcttcaaa	atggctaggattttgtcaaggcga
52	tgaagattgttatgttggttcatgac	catagagatcatcaatattcattcgt
56	tcgtttttaccccactctgttttaaaca	gtctgttgctaagtgctgctagtgggtt
60	tgaccatacaatatagctactcactattta	ggttatcaaaccagtaattttgtagttgtt
64	gtagtgctgtcatttacgaaccccgttaattg	ccataatgatgccttcgtacataaaaggaagt
68	gactggaataacaaataatgctaccttagctata	ttctttggtgtccatgtgtgatacttagtaacta
72	gtttttatacttaactagggattgtacattatctac	tactcctactaagtctttgaaagataacctttctcg
76	gaacaacaccatacttcttgccacgatgtttaacagtt	tccgtagttgctattgttcgtgaggatttgatcgacca
80	gttcattcatagcccatataccaccgttatacatccagtt	agcccaatctcctacaatcataaactggtcatgatttcca
84	gattatataagaatttagctagaacttgtagattctgcgtgc	ctcggtcaattaaatcccctacacatacaacgcaatctttct
88	gtagttaatgcaaggatagtaaagactatcattaaaacgaatac	cactagacttattttccagatacctagcagcatcatagtgaata
92	gcaattaatgtaaccaatccaggctttgcagctataatatcttgac	gactaagtgccttatcaagctgattaattttcttgtctagatctgc
96	gagaagagatctttttagtagctactgcaagatcgcctgctagagtct	caaccttattaactaagtgataagcaccgaatcctatgcctatcgcta
100	gatattttaacgcctaaatggttatcgcttcttactttttggttacaag	ttgtcattatttgcttttgctagtaagttgacttagtgtcaatcttatgt
104	gttttcaaactcgtgctccttgttgtctatacgaaggtgtttaatgaaccat	ctctctgttattctttaggaaacccattaaactcttcgatgactgctagagt
108	gaccgtggtatcagtattcctggctgtccgattggtattgattcagattctgaa	gtggatttgaaccactgtagtcgctccgtatgaaggaggtgcataaccactctg
112	gaggtacttgacgaaccacaccataaatcatcttatatcggatgcctctagctggt	cagattctagtgaaggtaaatctttaaatactctagctagtaatctatgtaattcg
116	gttatgtcgtctaaagacaattctggttttatcaaccttcctgtaggttttggcttac	caaacggtgtactctagccaattgagctaatcggagaaaattatttcttcagccacaa
120	gctttgagttttttcaatagctgcattaagttctttaagaaactcttttcgtacattaaga	tggtcggaaccacaattaccacacactttcttacctgttttagagtagtaatcttgatca
124	gtatcagacaatcctagttctttagaggcacgaatccaggaatatttagatacccagtactc	tgcaatagcctggttgttacgagcttcacgatctgcttctgcttgtttaaccttctgctgtg
128	cagtttgcaattgtgcttgaattttagcatcaaacaaatcttgtgctttaggtacagacttacc	atctagagcctgatcctgagtacctgcgttaatacggttaatcggtgccttagaaccatcaaac
132	catgtcaagacttgaccatccaggcaaaatttttactggaaatcatgaaatgttaactaaaaccat	gctgctgcgaggtaagatgcttgggctttcgtctaccgatttactagcttttaaattattactcta
136	ctagagccaactccataatattttgggccatagagtaagcagcatagttagagccgagtttattagta	tttatttatcacatctcggccatttcagcttcagtaataactgtgttacacaatgcatctacagcttc
140	cacctatcattgatatgggagtgtaaatatcgtgtgtaatatcatctggcagtggacgatcagcacgaat	tgactaggccattctacaccaatgtgcataaatgcattacgccccacaaagtacaagtattcttctagaa
144	caggagctgtgctggtgctttagccactgcttcgtctaccagatctttgtcaaactgctggaacataatctg	ctgatttcagtttcagtccaaaattgagagcgagctaataacgccagcctttctagctctggatgggctact
148	ctgtttatctgtgtttctaagtagtgttgggtctttggttatagagagaattaaagaatctcgatactctatcg	tgcttcttcaccagctcctccaccattcgcagtgttgaagtttgcgtagtaccaagtcttccaaccatatttaa
152	ccaggaatgatttcattctcaaacttggtgtagtcatagtacatattagtactgatagactggctgaagaatttct	tatttgagaaacggaaagaattaccatacaagctctatcctattaggggtagaatatcaatgttgtctcaattagt
156	cagttccgtgatatgatggaacgggcgtgtaggttgagtaatttcaacacaaaggtttgtcatacggatcggatcaag	ccatagtaatggcagaccaacttacatcaactgttttacaaccatactctgcccaaccattgagtttttcaggagcaa
160	ctcaacggaaatattctgagcttcatcgacaattacataggagttgttgaaagttctgccacgagcatgttctatagcca	tattgacatctgcccttgaataagctataatagtagtcttagttagagaaggagggtataatgaatcgcaatgatgaact
164	cactggaatgggttattaattatgctgctagtaaggttcattacttcggtattaatactccagtggattcctgtggtaaatg	actccaaaatattatctaggggttcaagagaggaatcatataatgcgtaggctaattattattagtggtgcaggtttgagtg
168	ctattcttaaccgtactgctgcgaaacttatggtagttaactactatgatagtgccctagcatcgaaaccaggctatcaaggta	taagctgtttatgcctaataaacaccgtattgttgttaactataaaaccagtgaatctcctggctttcaagttaatgaacatta
172	ctgaattcggaaaggtaaatccaaaccgtgcatatttagctcgttatataggcagagttttatatgggggtgcgaacgaaaagagc	ttatcatcttgagtctattaaagatgtaacagttgagcatgtcgaacaatatatcaaagataaaccgaatgatcgagctgcattct
176	ctcatctggcagaagcaatccgagctatcactaagtaattttagataaatccaccaacaaaaggaactatagctatgactaaagctga	aaggagggggaacaagtaagattaacagtatctgaatttcgtgggaatctatacatgggtattcgttactggcttcttgatattaatg
180	ctagagatcggttgccagattcgagtgattcgtgctggtggtgtaatcccgtgcatagtagagcgagtatacgattaatacataaccctt	tgaaattcctagacttagaataaaattatgaaggttgttatatctaataaagcctatttcaagcccgatgatgaactttgggattattgt
184	ctataaagcctggctaaatttccccacaaggcaaggatgtaatcgaattaccgtggaaattctcaaagggtattaagggtgctaaagctgagg	gaaatttgatgaaaatcgaaattccaacacaatgtccctcttgtggttctaagctagatcttgtcaacggacaattattctgtcgtaataag
188	caaacaatactagaagctcttacacagcgtggtgttacaacctatgaaattataggagcaacccacttagatgaccgattaaaaattcaggaag	gattagccagaactagagctagaatgtttaacggtggtgaagagcttttcgataacttcgttaaacaatacacaatggctaagatgagtgattt
192	cgattgtactaatacagataatgatcagttaaaattactactaactatttataatcttggtattatagaaggttgtagaagtgaataatagaaaaa	ataaattagttgctagatatggggataaagttacagtagctttacttgactatattaaccaaacccgacttccagattctaaaaccattgatatgt
196	cgagatgctaaagagtacattgagacttacttcggtcagttcccacagcttaagcgttggattgataagtgccacgatcagatcaagaatcatggatt	attttaatctgttgtctcaatccagatcatgacgataaacacccaagtttgcgtattgaccctgaaacaggtattatgcactgcttgagttgtggctt
200	ctgtggtgttcagggtgaagaaattcgttctggatttaatgcaatcattcagtctgcttcttctgatagtctccttttaggtgctgtagatgcagataat	gtatttatcaacatgagaaatgaacccgataaatacccagacttccactccaacatcgcacacatggtgtttaagctgcaatgcgaaccccgtgatgtta

Lambda

Increment	Left Stuffer Sequence	Right Stuffer Sequence
4	tg	ac
8	tcaa	aaag
12	taggcc	agcgag
16	tcggcgtt	ttccggaa
20	tgtgaatggg	cggatgctaa
24	tcagcgcaacac	ccttatctggtt
28	taaaaaactaccgt	gaaaagtcggtgga
32	tctggacccgtgatgg	cattctctggttttcg
36	tcatccggtgaagagatt	gagccacctgacagtgtg
40	tgtgggaggcgaaaattggc	gaacgtccggatgctgaagt
44	tgccacgacgatgaacagacgc	tgctgcgtgtggatgaggccat
48	tctgacctttcacatctggacagc	gtacagcccgttcagcacctgggt
52	tgcaggtcgaaaaatgggtggatggc	aggaaagcaatactctgggacacgta
56	tattacgcccgtgccttatccggagagg	atgaatgacgcgacaggaagaacttgac
60	tcggtgagacgtgggaggcgaaaattggcg	aacgtccggatgctgaagtgatggcagagc
64	gagcggaaagagcattattcagcgcccgttcc	tgaccgtgtggcttacctgaccgccggtatcg
68	gactcccagctggaccgctacgaaatgcgcgtat	ggggatgggggccgggtgaggaaagctggctgat
72	gtccaagggaccgagtgaaagtgtggatgcagccct	gttgcccaactttacccgtggcaatgcccgcgcaca
76	gtgttcgatccgaaaggctgggcgctgttccgttcctt	caaagccgtcaaggacaacgtggatacccgtcgtggct
80	gacattgttacactgtggaggagtccatgacgaaagatga	actgattgcccgtctccgctcgctgggtgaacaactgaac
84	gtgagcagtcaggtggcgtgatacgtggtgtttttgatgacc	ctgaaaatatcagctatgccggacagggcgtgcgcgttgaag
88	gtatggtggccagcggctatgactaccggcgcgacgatgatgcg	ggcttgtggagttcagccgatctgacttatgtcattacctatga
92	gcaggaagtcgttaccacctggcccacggagccaatttctcatgct	gaaaacgtggtgtaccggctgtctggtatgtatgagtttgtggtga
96	gatgcgtgatggtggcctgttctccggaggtggacgatgaagaccttc	cgctggaaagtgaaacccggtatggatgtggcttcggtcccttctgta
100	ggtgacgagtgcggttatagcggtccggctgtcgcggatgaatatgacca	gccaacgtccgatatcacgaaggataaatgcagcaaatgcctgagcggtt
104	ggaagtccgcctgctggttcagatacaacgtaacggtggctgggtgacggaa	aaagacatcaccattaagcgcaaaacgacgtcgcagtatctggcctcggtgg
108	gtcgaggattgagctgacgccgggctattttcagataaccgccacgccgcatct	tgccgtttatgacccgacggtacagtttgagttctggttctcggaaaagcagat
112	gacagcgtgaaagcagtgtggactggccgtcaggtacccgtactgtcaccgtgacc	gatgaccatccttttgatcgccagatagtggtgcttccgctgactgacgtttcgag
116	gcacttgcggtgacagtcactcaggcccgtgcggaaggtggacatggtacgtttacgg	tgggctattttcaagtgaaaccgggtacattgccgtcgttgttgtcgggcggggatac
120	gtggttcgccgcgtcgaaaaagagcagcacagtgatgaccggggaggatacgtttcacta	tgagagcctgcgtggacgttatgtgagcgtgatggccggaccggttttacaaatcagtaa
124	cgtaagacggaaatcactcccgggtatatgaaagagacgaccactgccagggacgaaagtgc	aatgcggcatacctcagtggcgtggagtgcaggtatacagattaatccggcagcgtccgtcg
128	ctgatgaagccgggcgttacagcatggatgtggagtacggtcagtacagtgtcatcctgcaggt	tgacggttttccaccatcgcacgccgggaccatcaccgtgtatgaagattcacaaccggggacg
132	ctggaagtgcggtatcagcatcgcagagcaaaagtgcggcagaagcggcggcaatacgtgcaaaaa	attcggcaaaacgtgcagaagatatagcttcagctgtcgcgcttgaggatgcggacacaacgagaa
136	ctcggaaaacaccgtcaaaaacattgcataattctatattgtgaggcttgcataatggcattcagaat	gagtgaacaaccacggaccataaaaattttctgctggccggaactaatgaatttattggtgaaggtga
140	cctggaagcctggaagaagtatcgggtgttgctgaaccgtgttgatacatcaactgcacctgatattgag	tggcctgctgtccctgttatggagtaatcggttttgtgatatgccgcagaaagcttgtatgaaataacgt
144	cttcaggagaataatggaagttctatgactcaattgttcatagtgtttacatcaccgccaattgcttttaag	actgaacgcatgaaatatggtttttcgtatgttttgagcttgctgttgatatttctaaagtcggtttttttt
148	catttttgattattatttgaatcaattccaattacctgaagtctttcatctataattggcattgtatgtattgg	tttattggagtagatgcttgctaatctatgtattggtttattggagtagatgcttgcttttctgacgcatagct
152	caattaatgtttgaatgtgataaccgtcctttaaaaaagtcgtttctgcaagcttggctgtatagtcaactaactc	ttctgtcgaagtgatattttaggtatctaccagttttagacgctctttaatatcttcaggaattattttattgtc
156	ctaaataagttattctcctggcttcatcaaataaagagtcgaatgatgttggcgaaatcacatcgtcacccattggat	tgtttatttgtatgccaagagagttacagcagttatacattctgccatagattatagctaaggcatgtaataattcgt
160	catgttactgttcttgcggtttggaggaattgattcaaattcaagcgaaataattcagggtcaaaatatgtatcaatgca	gcatttgagcaagtgcgataaatctttaagtcttctttcccatggtttttagtcataaaactctccattttgataggtt
164	cacgtcgtatgcaggaatgtgctgcggctggctggtgaacttccgatagtgcgggtgttgaatgatttccagttgctaccga	ttttacatattaattgcacgatttacatattaattcgatgagagaatttgtaccacctcccaccgaccatctatgactgta
168	caccggattttgtaaaaacagccctcctcatataaaaagtattcgttcacttccgataagcgtcgtaattttctatctttcatc	atattctagatccctctgaaaaaatcttccgagtttgctaggcactgatacataactgttttccaataattcgggaagtcattc
172	cgtttcagatttcgttcaataaattctgactgtagctgctgatagcttgcggttgaactatatttccttataacttttacgataga	gtttctttgagtaacttcactcaagtgcttccctgcctccaaacgatacctgttagcaatatttaatagcttgaaatgatgaagag
176	cgaaatttgcatatacccattgctcacgaaaaaaaatgtccttgtcgatatagggatgaatcgcttggtgtacctcatctactgcgaa	cacaaaatggacattttcacctgatgaaacaagcatgtcatcgtaatatgttctagcgggtttgtttttatctcggagattattttc
180	cctttgtcagcttaccaactactaagcttgtaggctcaagagcgtgtgtcctgtcgtaggtaaataactgacctgtcgagcttaatattc	tatattgttgatttttttagaatggtttcataagaaagctctgaatcaacggactgcgataataagtggtggtatccagaatttgtcactt
184	cgtaggactttccacatgcaggattttggaacctcttgcagtactactggggaatgagttgcaattattgctacaccattgcgtgcatcgag	taagtcgcaagcagatatttctggatattgtcataaaagaatttactgaatttatcatcgtccacttgaatctgtggttcattacgtcttaa
188	ctgagatcgcctagtgattttaaactattgctggcagcattcttgagtccaatataaaagtattgtgtaccttttgctgggtcaggttgttctt	taggagaggtatagttaaatcactgaatccgggagcactttttctattaaatgaaaagtggaaatctcagatatctggcaaaccattatacaca
192	ctctgaatagctttaagaaggttatgtttaaaaccatcgcttaatttcgtgagattaacatagtagtcaatgctttcacctaaggaaaaaaacatt	tcagtaacaaaggaatagtcattccaaccatctgctcgtaggaatcggttattttttttctactgcaggaatatacccgcctctttcaataacacta
196	ctcaatttatttggcggcaacacaggatctctcttttaagttactctctattacatacgttttccatctaaaaattagtagtattgaacttaacgggg	catgcttccttttggataacccactgttattcatgttgcatggtgcactgtttataccaacgatatagtctattaatgcatatatagtatcgccgaac
200	cacttcgtagccatttttcataagtgttaacttccgctcctcgctcataacagacattcactacagttatggcggaaaggtatgcatgctgggtgtgggg	caagaagacaaaaatcaccttgcgctaatgctctgttacaggtcactaataccatctaagtagttgattcatagtgactgcatatgttgtgttttacagt
